# Clusters of Spin Valve Sensors in 3D Magnetic Field of a Label

**DOI:** 10.3390/s21113595

**Published:** 2021-05-21

**Authors:** Georgy V. Babaytsev, Nikolay G. Chechenin, Irina O. Dzhun, Mikhail G. Kozin, Alexey V. Makunin, Irina L. Romashkina

**Affiliations:** Skobeltsyn Institute of Nuclear Physics, Lomonosov Moscow State University, Leninskie Gory 1/2, 119991 Moscow, Russia; kvyvg-george93@mail.ru (G.V.B.); irina.dzhun@gmail.com (I.O.D.); kozin@srd.sinp.msu.ru (M.G.K.); avmtchem@mail.ru (A.V.M.); irom@srd.sinp.msu.ru (I.L.R.)

**Keywords:** spin-valve sensor, magnetic label, inhomogeneous magnetic field, Stoner-Wohlfarth model, giant magnetoresistance, Wheatstone bridge

## Abstract

Magnetic field sensors based on the giant magnetoresistance (GMR) effect have a number of practical current and future applications. We report on a modeling of the magnetoresistive response of moving spin-valve (SV) GMR sensors combined in certain cluster networks to an inhomogeneous magnetic field of a label. We predicted a large variety of sensor responses dependent on the number of sensors in the cluster, their types of interconnections, the orientation of the cluster, and the trajectory of sensor motion relative to the label. The model included a specific shape of the label, producing an inhomogeneous magnetic field. The results can be used for the optimal design of positioning devices.

## 1. Introduction

Magnetic field sensors based on various physical effects play an important role in modern science, industry, and everyday life. A number of reviews have been published on applications of sensors in different spheres. A review of applications of modern, highly sensitive magnetometers can be found in Grosz and Haji-Sheikh [1]. A comprehensive review focused primarily on biological and medical applications of different types of magnetic field sensors is given in Murzin et al. [2]. Among the basic principles of sensor functioning, magnetoresistance (MR) has the widest scope of development and applications. A roadmap of this type of sensor was recently published [3]. Sensors employing the anisotropic, giant, and tunnel magnetoresistance effects are becoming more and more competitive because they can offer a variety of attractive properties suited for specific uses. MR sensors offer high sensitivity sought for biomedical applications [2,3,4,5,6,7]; high mechanical flexibility, small size, and robustness for wearable and portable devices [8,9,10,11]; low power consumption and small physical size, for position sensing [12,13,14]; low cost and mass production for large-scale, non-destructive evaluation and monitoring systems [15,16,17,18]; high accuracy and stability for navigation and transportation systems [19,20,21,22]; and high radiation hardness for space applications [13,14,16,21,22,23].

Spin-valve (SV) sensors are a type of magnetic field sensor based on the giant magnetoresistance (GMR) effect, discovered by two research groups led by Nobel Prize Laureates for Physics in 2007 Albert Fert [24] and Peter Grünberg [25]. Different aspects of the nature of the GMR effect and applications of the effect in magnetoresistive sensors are presented in [26]. SV sensors are a specific type of GMR sensor. The output signal of a SV sensor depends on the applied magnetic field via the angle ϕ between magnetizations of the free and pinned ferromagnetic layers of SV structure by (1-cosϕ) law [27]. This law follows from the Stoner-Wohlfarth model of coherent rotation of free layer magnetization [28]. Despite the fact that this model oversimplifies the real picture, it captures the main features of the phenomenon and allows the calculation of GMR as a function of an external magnetic field R_GMR_(H).

From the experimental side, the angular variation of the GMR effect was found to be very close to the relation R_GMR_(ϕ) = R^0^_GMR_ (1 − cosϕ)/2 in the current-in-plane (CIP) geometry for various systems, where R^0^_GMR_ is the amplitude of the GMR effect [27,29].

Usually, single sensing elements are combined in groups or clusters, the most familiar being a Wheatstone bridge (WB) with one to four active arms in the bridge. The inactive elements are either deactivated by magnetic shielding, or made from nonmagnetic material with close electric resistance.

In the present work, we consider linear movement of a macroscopic ((sub)-millimeter range) sensor in the form of the Wheatstone bridge with one or two point-size sensing elements in the inhomogeneous field of a magnetic label. The magnetic label has macroscopic (millimeter range) dimensions and the form of a cuboid (parallelepiped with square base), cylinder, or other shape that allows calculation of the components of a magnetic field in close vicinity to the label, where it differs from a dipolar field. We calculate the signals of the unbalanced bridge for parallel and perpendicular orientations of sensing element magnetization relative to the direction of linear movement of the bridge parallel to the y-axis of the Cartesian coordinate system with magnetic label in the origin. All sensing elements are deposited in the same plane on the surface of a substrate. We investigate the dependence of the signal on the size of the bridge and on the nearest approaching distance between the bridge center and the label along the trajectory, referred to as the impact parameter.

Despite the fact that the Wheatstone bridge configuration is a common subject in the literature [30], to the best of our knowledge, our study is the first to consider in detail the position sensing of a magnetic object using WB. The present results can be used for positioning magnetic objects in 3D space, and for label monitoring with a network of bridge clusters.

## 2. Materials and Methods

### 2.1. Simulated Sensors and Magnetic Label

**Figure 1:**
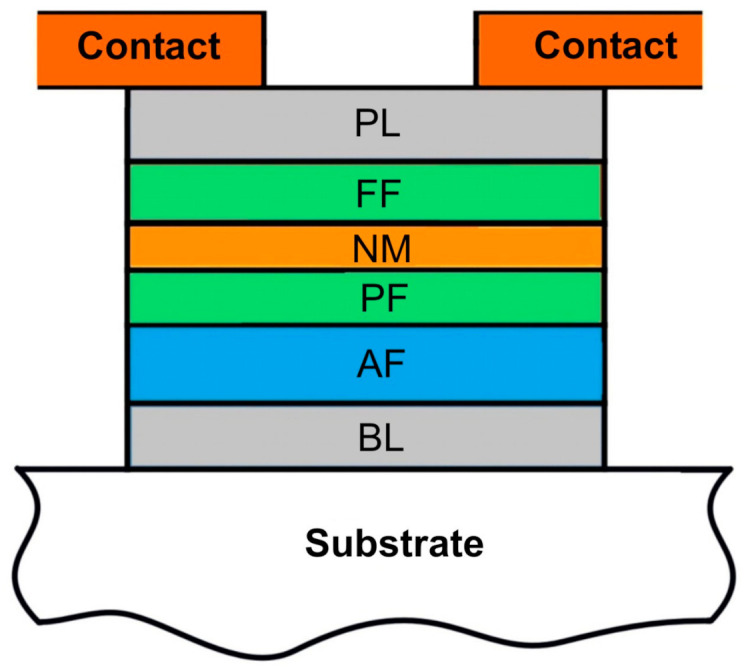
Schematic representation of a SV layer structure: BL denotes a buffer layer; AF, antiferromagnetic layer; PF, pinned ferromagnetic layer; NM, non-magnetic layer; FF, free ferromagnetic layer; and PL, protective layer.

The simulated system consists of a magnetic label and a cluster of SV sensors. In our calculations, we assume the magnetic label is in the form of a cuboid with dimensions of 2 × 2 × 3 mm^3^ with the remanence B_r_ = 0.4 T, which corresponded to the label parameters in our experiments. The simulated clusters of SV sensors are 4 SV elements configured into a rectangular 4-arm Wheatstone bridge. The SV elements are multilayer structures typically deposited in CIP geometry. A schematic representation of the layers of the SV structure is shown in Figure 1. The layers FF/NM/PF/AF are the most important part of the SV structure, where FF is a free ferromagnetic layer; NM, a non-magnetic layer; PF, a pinned ferromagnetic layer; and AF, an antiferromagnetic layer. It is assumed in the calculations that PF has a strongly pinned orientation of magnetic moment, making PF not sensitive to the external magnetic field. In contrast, the FF layer is made of a soft material with magnetic moment that rotates under the influence of an external magnetic field. The most important magnetic parameters of the FF used in calculation are the saturation magnetization Ms = 100 kA/m and uniaxial anisotropy coefficient K_u_ = 200 J/m^3^, corresponding to Permalloy, used in our experiments [31]. The bridge is normally deposited in a single deposition run; therefore, all 4 SV elements were identical, providing the balance in the output signal in the absence of the magnetic field. In addition, 2 or 3 SV elements were shielded so that they were insensitive to the magnetic field, leaving 1 or 2 sensitive SV elements in the bridge.

### 2.2. Simulation Model

A permanent magnet label creates an inhomogeneous 3D magnetic field. The method of calculating the 3D components of the magnetic field of the label, **H(R)**, was reported earlier [32]. A similar method was used in our previous papers [33,34] to calculate the fields of permanent magnets of several forms (parallelepiped, cylinder, etc.). We chose the Cartesian coordinate system connected with the label placed at the origin and with magnetic moment oriented along the z-axis. Instead of translation of the label, we consider the motion of the sensor along a straight-line trajectory in the yz-plane at a distance x_0_ from the origin and z_0_ from the xy plane. We follow the variation of the sensor signal during the motion along the trajectory, i.e., we study the variation of the signal depending on the sensor location relative to the label and on the relative mutual orientation of the sensor and the magnetic label. The results for orientations of 6 single sensors were given in Babaitsev et al. [33,34] are used here for clusters of SV sensors.

The single sensor is supposed to be a point-like object characterized by direction of uniaxial anisotropy in the free ferromagnetic (FF) layer and unidirectional anisotropy in the ferromagnetic pinned (FP) layer, defined by the direction of the magnetic field during the deposition of the layers. The orientation of the structure is characterized by the normal **n** to the plane of the multilayer structure of the sensor. The vector **M** is the magnetic moment of the FF layer; Ms is the value in saturation.

Here we assume that the Stoner-Wohlfarth (SW) model [28,35], which often is used to describe the processes occurring in SV, is also valid for the magnetic film. The model allows us to evaluate the reorientation of the magnetization of the anisotropic particle under the influence of an applied field. Applying the magnetic field in a direction different from the direction of the easy axis (EA), one can initiate a coherent rotation of the magnetic moment **M** in the FF layer of the SV. Further on, the model assumes that the rotation and equilibrium state of magnetization are achieved instantly. An in-depth consideration of signal features of a single SV sensor moving in a magnetic field of a label can be found in Babaitsev et al. [33,34]. In this paper, we study the signal of a cluster of sensors instead of a single SV sensor.

**Figure 2:**
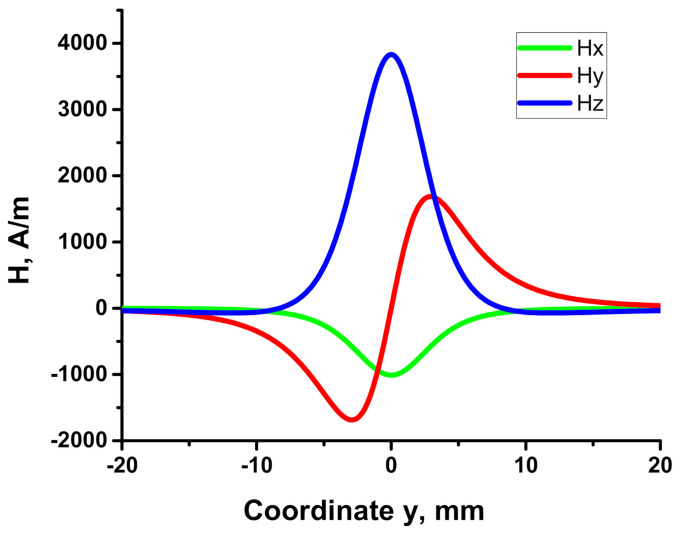
An example of y-dependence of the magnetic field components along the trajectory {x = −1, y = var, z = 6 mm} for a cuboid label.

The variation of magnetic field components along the y-direction for a cuboid label is illustrated in Figure 2. There are constant-sign dependences for 2 components of the magnetic field, H_x_ and H_z_, and an alternating-sign for another one, H_y_. Similar calculations can be made for magnetic labels in the form of a cylinder or ball.

### 2.3. Interaction of the Magnetic Label and the Cluster of SV Sensors

**Figure 3:**
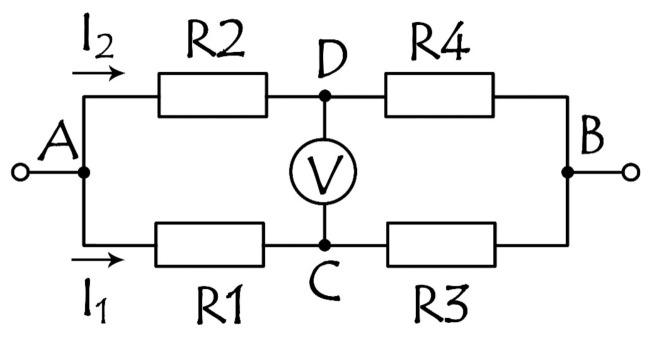
Wheatstone bridge circuit. A, B are input, C, D—output contact points, R1–R4 are resistances.

The simplest cluster is the Wheatstone bridge (Figure 3). The principle of operation of the bridge is based on the equalization of potentials of the middle terminals of 2 resistor branches connected in parallel, each of which has 2 resistors.

When powered by voltage, the bridge provides a differential output U_DC_ as a function of resistance change

(1) $$U_{\mathrm{DC}}=U_{\mathrm{AB}}\left(\frac{R4}{R2+R4}-\frac{R3}{R1+R3}\right),$$

where U_AB_ is a bias voltage, and R1–R4 are resistances in the arms of the bridge.

In our calculations the bias voltage of the bridge is 10 V, and the resistances of SV R_P_ and R_AP_ are equal to 20 and 22 Ohm (R_P_ and R_AP_—the resistances at parallel and antiparallel magnetization of free and pinned layers, respectively).

**Figure 4:**
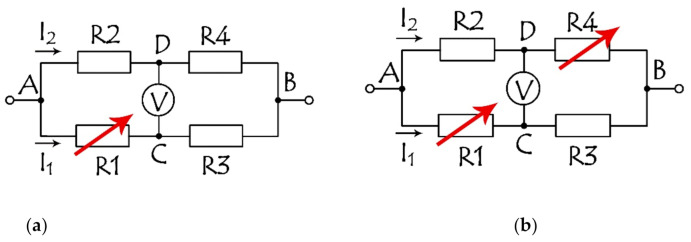
Bridge circuits with 1 (**a**) and 2 (**b**) sensing arms, marked with red arrows indicating structures that change their resistance.

Without a magnetic field, the SV bridge is balanced, and the signal from the bridge is absent. With a magnetic field applied, the signal appears and is recorded by the voltage change. The arms of the bridge are made of identical SV structures to exclude a misbalance in the bridge in a no-field environment and the necessity of adjusting the resistors and construct additional microcircuits for balancing the bridge. The identity can be easily achieved by the SV layer deposition in 1 cycle. Three or two arms can be shielded, making the bridge with one R1 = R + ΔR (Figure 4a), or with two R1 = R + ΔR1, R4 = R + ΔR4 (Figure 4b) sensitive elements in diagonal position. The output voltage as a function of the resistance of 1 or 2 sensitive elements in Figure 4 can be expressed, respectively, as Equation (2) or Equation (3).

(2) $$U_{\mathrm{DC}}=U_{\mathrm{AB}}\frac{\frac{\Delta R}{R}}{2\left(2+\frac{\Delta R}{R}\right)}$$

(3) $$U_{\mathrm{DC}}=U_{\mathrm{AB}}\left(\frac{R+\Delta R4}{2R+\Delta R4}-\frac{R}{2R+\Delta R1}\right)$$

## 3. Results and Discussion

**Figure 5:**
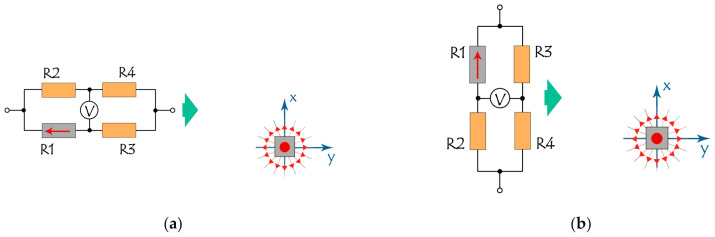
Mutual orientation and location of the moving bridge and magnetic label (grey squares). The direction of magnetization **M** for sensitive element R1 in the bridge (red arrow) is either parallel to **y** (**a**) or parallel to **x** (**b**), i.e., either parallel or perpendicular to the movement direction of the bridge (green arrow). Red point in the center of the gray square symbolizes z-direction of magnetization for the label. Red lines with arrows around the labels show schematic projections of the label magnetic field onto the xy plane.

Let us consider first a simplest cluster configuration with one sensitive SV element and the other three of four identical elements shielded, as shown in Figure 5 for two identical sensors with two different orientations with respect to the label. The plane of both sensors is parallel to the XY plane, but the directions of magnetization of the sensitive SV elements are parallel (Figure 5a) or perpendicular (Figure 5b) to the line of movement of the bridge, and both are perpendicular to the magnetic moment of the label.

**Figure 6:**
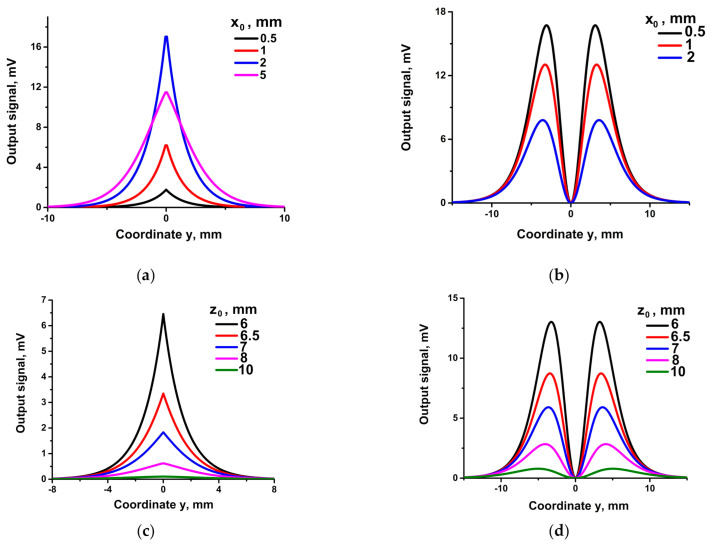
Output signals of the SV bridges with one sensitive R1 element as a function of the position along the y coordinate. Bridge orientations correspond to **M**||**y** (Figure 5a) on the left panel, (i.e., **a**,**c**) and **M||x** (Figure 5b) on the right panel,(i.e., **b**,**d**). Curves in (**a**,**b**) correspond to the trajectories of R1, respectively, {x = x_0_ = 0.5; 1; 2; 5 mm, y = var; z = z_0_ = 6 mm}, and in (**c**,**d**) correspond to the R1 trajectories, respectively { x = x_0_ = 1 mm, y = var, z_0_ = z = 6; 6.5; 7; 8; 10 mm}.

Representative results of numerical calculation of the response of such bridges are shown in Figure 6 (a,b—for different x_0_-coordinates and c,d—for different z_0_-coordinates of the R1 element).

The shape of the curves in Figure 6a,c (left panel) is a single peak, while in Figure 6b,d (right panel) the curves have two peaks. In addition, one can note that the output peak value varies in Figure 6a non-monotonously when the parameter x_0_ increases At first, the peak value increases (curves x_0_ = 0.5; 1; 2 mm), then decreases (curve x_0_ = 5 mm) at the same time that the width of curve x_0_ = 5 mm increases. The non-monotony may look somewhat surprising; the increase of x_0_ from 0.5 mm to 2 mm, and, hence, the distance from the label magnetic field decreases and should lead at first sight to a smaller rotation of **M** and smaller signal in R1.

The observed features can be interpreted based on consideration of the output signal of a single SV sensor in the field of a magnetic label reported previously. In particular, the case in Figure 4a corresponds to case 2.1 in Babaitsev et al. [33], when the magnetic moment of the R1 sensor has components **M** = {Ms, 0, 0} in the initial state. Non-monotony is due to two factors, besides the distance between the sensor and the label. (1) Due to a demagnetizing field vector, **M** can rotate within the plane of the sensor, i.e., in the xy plane in the case of Figure 5a, so during the movement in y-direction, **M** will have an in-plane component M_xy_ with M_z_ = 0. (2) Only a perpendicular to **M** component of the magnetic field, except H_z_, can cause the tilt of **M**. Variation of the H_xy_ (x_0_, y, z_0_) components of the 3D field of the magnetic label makes the observed feature of the output signal of the SV sensor a reflection of variation of the tilt angle ϕ = arctg (M_x_/M_y_).

Due to the initial orientation of **M** in Figure 5a, parallel to the y-axis, M_y_ = M_s_, the dominating rotation action is due to the y-component of non- monotony **H**. It is important to note that H_y_ keeps the sign on the trajectories 1–5 in Figure 6a,c, slowly increasing and decreasing when crossing the space plane {x, 0, z}. Therefore, the output signal is of a single-peak shape.

In contrast, the dominating rotational effect on M_x_ = M_s_ for the case of Figure 5b is due to the component H_y_ of the 3D label magnetic field. In this case, the H_y_ component changes sign when crossing the space plane {x, 0, z}, inducing the double-peak shapes of the curves of y-scan with trajectories {x = x_0_ = 0.5; 1; 2 mm, y = var; z = z_0_ = 6 mm} in Figure 6b and {x = x_0_ = 1 mm, y = var, z_0_ = z = 6; 6.5; 7; 8; 10 mm} in Figure 6d.

The bridge signal depends only on the R1 coordinate and does not depend on R2–R4 coordinates (i.e., it does not depend on the bridge size). If, instead of R1, we make another sensing arm of the bridge, then the signal polarity for R4, as the sensing element, will be the same as for R1 and of opposite polarity for R2 or R3_._

**Figure 7:**
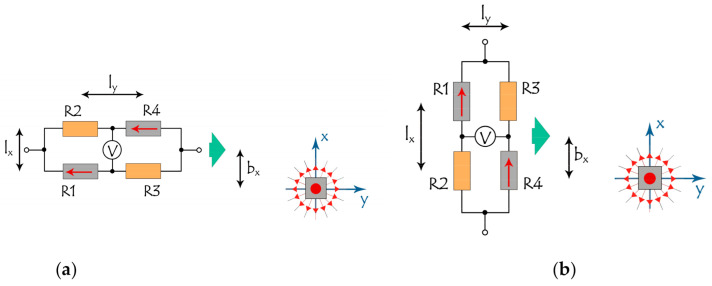
Mutual orientation and location of the moving bridge and magnetic label. l_x_, l_y_ are the distances between sensor elements along the x and y axes, and b_x_, b_z_ (the latter is not shown) are impact parameters (nearest approach distance) in x and z directions. The direction of magnetization for sensitive elements R1 and R4 of the bridge is parallel (**a**) or perpendicular (**b**) to the movement direction, shown by green arrow.

Configurations for a bridge with two sensing elements R1 and R4 are shown in Figure 7. In this case, the dimensions of the SV sensor and locations of the sensing elements within it are important. In Figure 7 the size of the sensor is characterized by distances between sensor elements along the x and y axes, l_x_ and l_y._ It is also convenient to introduce the parameter b_x_ and b_z_ (the latter is not shown), which, in analogy with the tradition in the field of atomic scattering, are called impact parameters in the x and z directions and are determined as the nearest approach distances between the center of the bridge and the label. These parameters play a role as coordinates of trajectories x_0_ and z_0_ in the bridge with a single sensitive element, considered above. Magnetic moments in the sensitive elements in the initial state are aligned in parallel, which is a normal practice in layer deposition and post-deposition treatment.

**Figure 8:**
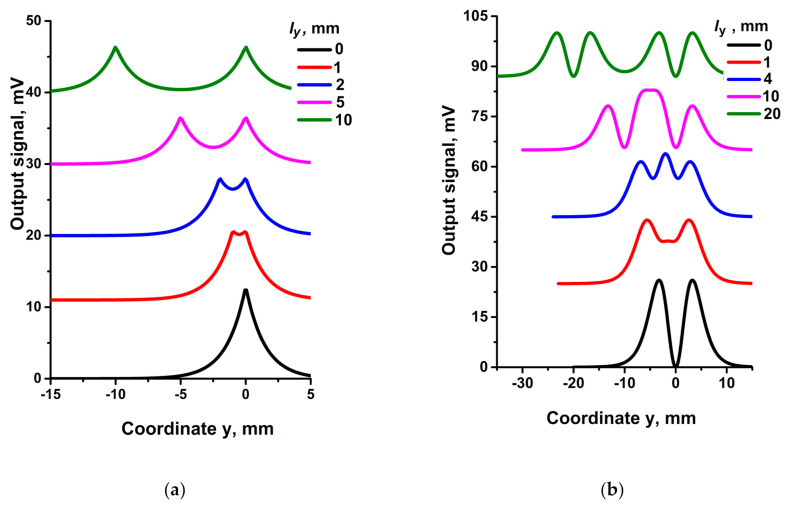
Representative responses of two bridges at different l_y_ = 0; 1; 2; 5; 10 mm ((**a**), bridge orientations correspond to Figure 7a), and l_y_ = 0; 1; 4, 10, 20 mm ((**b**), bridge orientations correspond to Figure 7b), at l_x_ = 2 mm, b_x_ = 0, b_z_ = 6 mm. R1 and R4 are the sensitive elements. Curves are shifted along the vertical axis for better readability.

**Figure 9:**
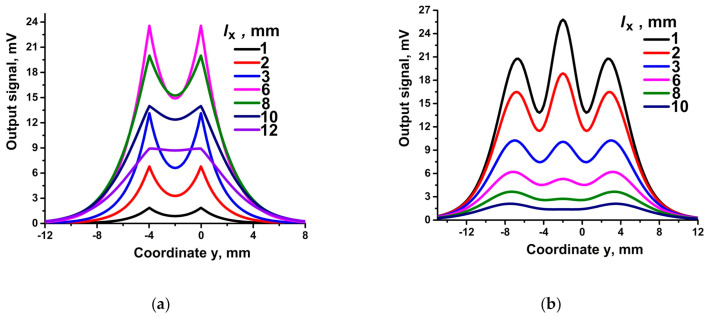
Response of two bridges at different l_x_ = 1; 2; 3; 6; 8; 10; 12 mm ((**a**), bridge orientations correspond to Figure 7a), and l_x_ = 1; 2; 3; 6; 8; 10 mm ((**b**), bridge orientations correspond to Figure 7b), at l_y_ = 4 mm, b_x_ = 0, b_z_ = 6 mm. R1 and R4 are the sensitive elements.

**Figure 10:**
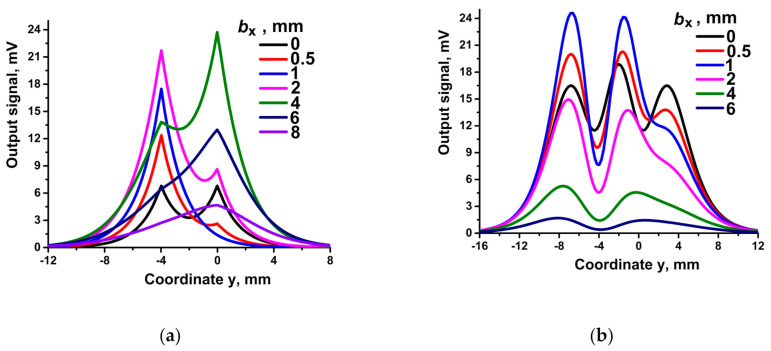
Response of two bridges at different b_x_ = 0; 0.5; 1; 2; 4; 6; 8 mm ((**a**), bridge orientations correspond to Figure 7a), and b_x_ = 0; 0.5; 1; 2; 4; 6 mm ((**b**), bridge orientations correspond to Figure 7b). For both orientations l_x_ = 2 mm, l_y_ = 4 mm, b_z_ = 6 mm. R_1_ and R_4_ are the sensitive elements.

The results of numerical study of the response of such bridges with two orientations relative to the label and the direction of movement are shown in Figure 8, Figure 9 and Figure 10. The left panel in Figure 8, Figure 9 and Figure 10 corresponds to the y-orientation of the magnetic moments M_y_ = Ms in the sensitive elements, Figure 7a, while the right panel relates to the x-orientation, M_x_ = Ms, Figure 7b.

Figure 8 illustrates the shapes of output signals for a set of the bridge size in y-direction, l_y_.

The output signals in Figure 8a are composed of two peaks in curves for l_y_ from 1 to 10 mm. The peak at y = 0 corresponds to R1 position opposite the label while the second one at y ≠ 0 corresponds to R4 nearest the label. Separation of these peaks is determined by the value of l_y_; at l_y_ = 0 the peaks merge.

The output signal in Figure 8b is composed of two pairs of peaks (curve for l_y_ = 20 mm). A pair of peaks at y = 0 corresponds to the R1 position opposite the label, while the second pair at y ≠ 0 corresponds to R4. Separation of the centers of these pairs is determined by l_y_. As l_y_ decreases, two pairs of peaks transform into three peaks (curves for l_y_ = 1 and 4 mm), which then merge into two peaks at l_y_ = 0.

Figure 9 illustrates the variation of the shapes for a set of the bridge size in x-direction, l_x_. In both panels in Figure 9a,b one can see that the curve shapes (two and three peaks) correspond to l_y_ = 4 mm (Figure 8), which determines the peak positions. In Figure 9a, as l_x_ increases, the height of peaks at first increases (l_x_ = from 1 to 6 mm), and then the height decreases while the width increases (l_x_ from 8 to 12 mm). In Figure 9b, the height of the peaks decreases and the width increases as l_x_ increases.

Figure 10 illustrates the dependence of the signal on b_x_.

The types of curves and peak positions are determined by bridge dimensions l_x_ and l_y_. At b_x_ = 0, the heights of the peaks (Figure 10a) are equal (curve for b_x_ = 0). As b_x_ increases, the height of the left peak increases and the height of the right peak decreases (b_x_ from 0.5 to 2 mm). Then, the situation reverses, accompanied by widening and blurring of the peaks. The trend of changes in the picture (Figure 10b) is similar.

In general, for Figure 8, Figure 9 and Figure 10 the dimensions of the bridge l_y_ determine the number of peaks (or pairs of peaks) and the distance between the peaks (the centers of the pairs of peaks), l_x_ determines the magnitude of the response, and b_x,_ the asymmetry of the response.

**Figure 11:**
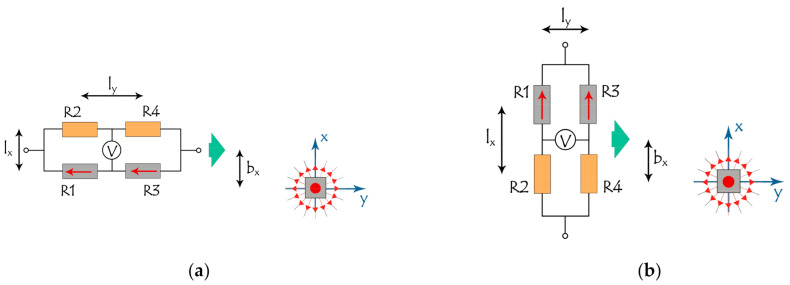
Mutual orientation and location of the moving bridge and magnetic label. R1 and R3 are the sensitive elements of the bridge, their direction of magnetization is parallel (**a**) or perpendicular (**b**) to the movement direction of the bridge.

Let us consider the situation of the bridge also with two sensitive elements but when these elements are R1 and R3 in adjacent positions. Two variants of this situation are depicted in Figure 11.

The output signals for the cases of the elements R1 and R3 connected in series (Figure 11a) and in parallel (Figure 11b) can be expressed, respectively, as Equations (4) and (5).

(4) $$U_{\mathrm{DC}}=U_{\mathrm{AB}}\left(\frac{1}{2}-\frac{1}{\frac{R+\Delta R1}{R+\Delta R3}+1}\right)$$

(5) $$U_{\mathrm{DC}}=U_{\mathrm{AB}}\left(\frac{R}{2R+\Delta R3}-\frac{R}{2R+\Delta R1}\right)$$

**Figure 12:**
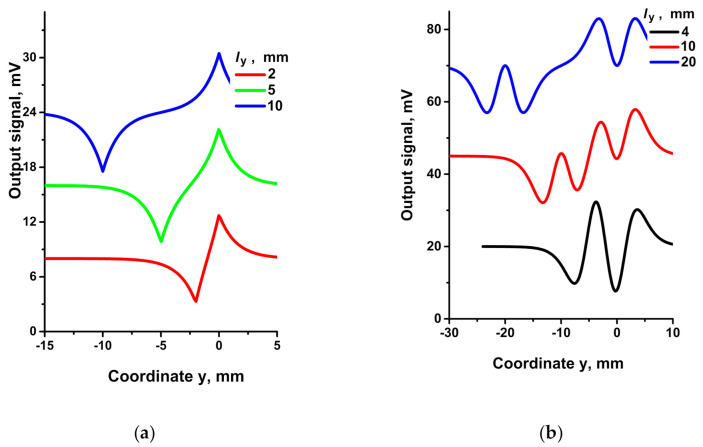
Response of two bridges at different l_y_ = 2; 5; 10 mm ((**a**), bridge orientations correspond to Figure 11a), and l_y_ = 4, 10; 20 mm ((**b**), bridge orientations correspond to Figure 11b), at l_x_ = 2 mm, b_x_ = 0, b_z_ = 6 mm. R1 and R3 are the sensitive elements with their direction of magnetization parallel (**a**) or perpendicular (**b**) to the movement direction of the bridge. Curves are shifted along the vertical axis for better readability.

The dependences of the output signal on the sensor position along y coordinate for a number of l_y_ parameters are shown in Figure 12.

In analogy with Figure 8a (left panel), there are also two peaks in Figure 12a (left panel). The peak at y = 0 (l_y_ = 2 to 10 mm) corresponding to the R1 position is positive. The second peak at y ≠ 0 is negative and corresponds to the R3 position. The distance between these peaks is determined by l_y_ for large l_y_. For small l_y,_ the peaks partially compensate each other (curve l_y_ = 2 mm), their heights diminish, and distance between them is already not equal to l_y_.

The dependences in Figure 12b (right panel) are similar to those in the left panel, but they concern not a single peak but pair of peaks. The positive pair of peaks at y = 0 corresponds to the R1 position, while the negative pair at y ≠ 0 corresponds to R3. The distance between the centers of these pairs is determined by l_y_ for large l_y_. For small l_y,_ the peaks partially compensate each other but in a more complex manner than in the left panel, which leads to curves of complex waveforms.

By connecting the sensitive elements of the bridge differently (Figure 7 and Figure 11), one can change the variability of the response of the measuring circuit.

## 4. Conclusions

Wheatstone bridge configurations of clusters of SV sensors moving in the 3D field of a magnetic label were considered as a sensor of the magnetic field of the label. Potentially, the WB network is a more favorable device than a single SV sensor, offering higher sensitivity to a weak magnetic field and to a weak space variation of the magnetic field. That is an important feature of WB-like sensors in many applications, including the localization and positioning of a moving magnetic object.

WB network output signals were considered for different bridge sizes, numbers of sensitive elements, and orientations with respect to the label.

We have demonstrated that the WB output signal, as a function of approach distance to the label, strongly depends on several factors. First is the trajectory of the sensor, i.e., the height and width of the signal strongly depend on not only the distance of the nearest approach of the sensor to the label, the so-called impact parameter, but also the position of the line of motion with respect to the geometry and orientation of the label.

The design and parameters of the WB sensor are a second factor. Here we considered WB-like sensors of rectangular design with four SV elements, balanced in a no-field environment, with one-of-four or two-of-four sensitive SV elements and the other SV elements protected from the influence of a magnetic field. We have shown that the shape and power of the output signal critically depend on the configuration of the sensitive and protected WB arms, the size of the sensor, the number (one or two) of sensitive elements, and spacing between the sensitive arms.

A third factor is the orientation of the WB sensor with respect to the label. We have demonstrated that the response of the sensor is crucially dependent on the initial orientation of the WB basic plane and magnetic moments of the sensitive elements within the plane.

Calculations provided new information on a WB-like SV sensor manifold response to the nonhomogeneous magnetic field of an object. This information can be used to design positioning devices for a variety of applications, including the localization and positioning of a moving magnetic object.

## Data Availability

Not applicable.
